# The analgesic effect of lumbar sympathetic ganglion block in patients with failed back surgery syndrome

**DOI:** 10.3389/fmed.2022.999655

**Published:** 2023-01-12

**Authors:** Jin Young Lee, Woo Seog Sim, Jiyoung Kim, Sungwon Yang, Hojun Ro, Chang Jae Kim, Sung Un Kim, Hue Jung Park

**Affiliations:** ^1^Department of Anesthesiology and Pain Medicine, Samsung Medical Center, School of Medicine, Sungkyunkwan University, Seoul, Republic of Korea; ^2^Department of Anesthesiology and Pain Medicine, Seoul St. Mary’s Hospital, College of Medicine, The Catholic University of Korea, Seoul, Republic of Korea

**Keywords:** spinal surgery, lumbar, sympathetic, ganglion, block

## Abstract

**Background:**

Persistent or recurrent lumbosacral pain is a common symptom after spinal surgery. Several interventions have been introduced for failed back surgery syndrome; however, their clinical efficacy, safety, and cost-effectiveness are insufficient. Sympathetic ganglion block has been selected for pain associated with the sympathetic nervous system. In this study, we compared pain and quality of life in patients with failed back surgery syndrome who responded and did not respond to lumbar sympathetic ganglion block.

**Methods:**

We included 84 patients diagnosed with failed back surgery syndrome who had lumbosacral pain and underwent lumbar sympathetic ganglion block between January 2020 and April 2021. The patients’ data were retrospectively analyzed; clinical outcomes were assessed before (T0), 1 week after (T1), and 4 weeks after (T4) lumbar sympathetic ganglion block. Based on the pain difference from T0 to T1, we categorized patients into two groups: patients with ≥ 50% pain reduction (responder group) and patients with < 50% pain reduction (non-responder group). Demographic, clinical, surgical, and fluoroscopic data were evaluated and compared. The primary outcome was pain scores and the EuroQol-5D score from T0 to T4.

**Results:**

Among the 84 patients analyzed, 41 (48.8%) experienced ≥ 50% pain reduction at 1 week after lumbar sympathetic ganglion block. Lumbar sympathetic ganglion block significantly improved pain at T1 and T4 compared to T0 in both groups. Lumbar sympathetic ganglion block improved the EuroQol-5D score at T1 compared to T0 in the responder group. The responder group had a significant decrease in pain at T1 from T0 and T4 from T0 and a significant decrease in the EuroQol-5D score at T1 from T0 compared with the non-responder group. Coldness of the leg over time did not differ between the groups. No serious adverse events occurred in either of the groups.

**Conclusion:**

Lumbar sympathetic ganglion block may improve pain at 1 and 4 weeks in patients with failed back surgery syndrome. Patients with ≥ 50% pain reduction at 1 week showed simultaneous improvement in quality of life and pain reduction at 4 weeks.

**Clinical trial registration:**

https://cris.nih.go.kr/cris/index/index.do, identifier KCT0007236.

## 1. Introduction

Chronic low back pain is one of the major causes of disability and leads to socioeconomic burden and psychological and lifestyle pressures. Failed back surgery syndrome (FBSS) is defined as persistent or recurrent pain, mainly in the region of the lower back and legs, even after technically anatomically successful lumbosacral spine surgeries (1). The incidence of FBSS is reported to be between 10 and 40% (2). It is reported to occur in up to 19% of cases after microdiscectomy and 25.5 and 40% of cases after laminectomy (3, 4). FBSS has multiple etiologies, including surgery adjacent lesion to the disc or facet area, persistent or recurrent neural compression, neuritis, fibrosis, hardware pain, and psychosocial factors (5–8). Patients with FBSS have higher pain levels, lower quality of life, and greater disability than patients with other chronic pain conditions such as rheumatoid arthritis, osteoarthritis, and complex regional pain syndrome (CRPS) (1, 2). In FBSS, neuropathic pain is a predominant pain-generating mechanism; however, there is no established guideline for the best treatment options (1). With conservative management, several interventions have been introduced for FBSS. Medial branch block and radiofrequency neurolysis, sacroiliac joint block, epidural steroid injection, and percutaneous epidural adhesiolysis have been found to improve pain, quality of life, or the degree of functional impairment with limitations (2). In FBSS, epidural fibrosis, instrumentation, and anatomical alteration after surgery can negatively affect the outcomes of epidural interventions. Input from the sympathetic ganglia is involved in various pain conditions (9). During nerve injury or tissue inflammation, the sympathetic nervous system may mediate pain by abnormal activation of alpha-adrenergic receptors of primary afferents, or direct interaction between efferent sympathetic fibers and primary afferent neurons during regeneration and sprouting (10–12). Blocking sympathetic neurons interrupts the positive feedback circuit and decreases central sensitization (9). Lumbar sympathetic ganglia are located at the anterolateral side of lumbar vertebrae *via* forming a synapse from pre and post-ganglionic fibers (13, 14). Lumbar sympathetic ganglion block (LSGB) is indicated for diagnosis and treatment for painful conditions including CRPS, herpes zoster, phantom limb, diabetic neuropathy, or vascular pain of the legs (9, 13). Patients with FBSS experience various natures of pain, including somatic components by neural injury and neuropathic components by nociceptive pain transmission from the disc and ligamentous tissue entering the sympathetic trunk *via* rami communicants (15). However, no study has examined LSGB outcomes for FBSS. Therefore, in this study, we compared pain and quality of life in patients with FBSS who responded and did not respond to LSGB.

## 2. Materials and methods

### 2.1. Study participants

We retrospectively reviewed the electronic medical records of 87 patients with FBSS who had lower back and leg pain and underwent LSGB between January 2020 and April 2021 at two tertiary care hospitals in Seoul, Korea. The enrolled patients ranged in age from 32 to 86 years old. The inclusion criteria were as follows: (a) > 6 months from lumbar spinal surgery; (b) a primary diagnosis of lower back pain radiating to the lower limbs; (c) cross-sectional imaging (either computed tomography or magnetic resonance imaging) of the lumbosacral spine in patients diagnosed with spinal stenosis or herniated nucleus pulposus after lumbar spinal surgery; and (d) insufficient pain control 1 month after lumbosacral epidural block, medial branch block, facet joint block or sacroiliac joint block. The exclusion criteria included neoplastic, peripheral vascular diseases, or failed lumbar sympathetic block (16). The lesion level for LSGB was chosen based on clinical manifestations, physical examination, and a review of imaging (16). Lesion severity was categorized as one of three different degree levels (mild, moderate, and severe) by reviewing imaging data (16). This study was approved by our departmental ethics committee (KC20RISI0917, SMC 2022-04-036) and registered with the Clinical Research Information Service of the Korea National Institute of Health (ref: KCT0007236).^1^

### 2.2. Clinical procedures

**FIGURE 1:**
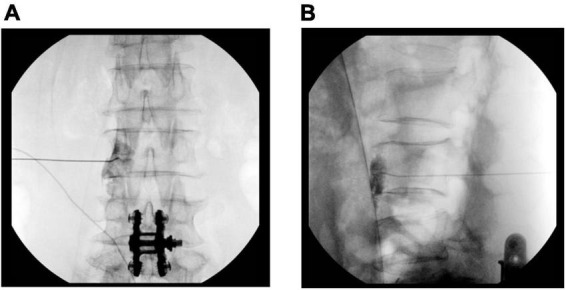
Fluoroscopic image of the lumbar sympathetic ganglion block. **(A)** Anteroposterior view of contrast media spread. **(B)** Lateral view of contrast media spread.

All procedures were performed under fluoroscopic guidance and were standardized. A physician determined the block level and side. Patients were placed in the prone position, and anteroposterior and lateral view images were obtained using a C-arm (OEC series 9800, GE Healthcare, Chicago, IL, USA) to ensure the proper entry site. Following the aseptic preparation and application of 1% lidocaine, a 21-gauge Chiba needle (Tae-Chang Industrial Co., Seoul, Korea) was advanced to the anterolateral border of the vertebral body. Aspirations were routinely performed to assess the presence of blood. When the needle position was confirmed by fluoroscopic imaging, an aspiration test was performed, and a contrast medium was injected. We assumed a successful block, which the lumbar sympathetic ganglion spread when the contrast medium was shown to form a line at the anterolateral margin of the vertebral body in the lateral view. The target point was confirmed with the anteroposterior and lateral view (Figure 1). When the contrast media was observed to be out of the margin of the vertebral body in the psoas muscle or spinal nerve in the anteroposterior view or out of the anterolateral margin of the vertebral body in the psoas muscle or spinal nerve in the lateral view, the needle was re-positioned to find the correct position. Then, a total volume of 10 ml of 1% lidocaine was injected on a single side per one level. The injection distance from surgery was defined as the intervertebral level from the surgery range. Fluoroscopic images were analyzed by two physicians who assisted with the procedure. Following the procedure, the patients were observed for any adverse effects and were discharged. Pain and coldness of the leg were scored using a numerical rate scale (NRS; ranging from 0 = no pain to 10 = absolutely intolerable pain). The EuroQol measure of health outcome (EQ-5D) was used to assess quality of life by scoring five dimensions of health (mobility, self-care, usual activities, pain/discomfort, and anxiety/depression) on five levels (none: 1, slight: 2, moderate: 3, severe: 4, extreme problems: 5) (17). The pain score, EQ-5D score, and coldness of the leg were recorded before the block (T0), 1 week after the block (T1) and 4 weeks after the block (T4).

### 2.3. Responder vs. non-responder groups

We defined the responder group as patients who showed ≥ 50% improvement in pain at T1 from T0 and the non-responder group as patients who showed < 50% improvement in pain at T1 from T0.

### 2.4. Statistical analysis

All data were analyzed using SAS 9.4 (SAS Institute, Cary, NC, USA). Data are expressed as the mean ± standard deviation (SD), median (interquartile range), or number (proportion), as appropriate. Demographic data for the two groups were compared using the Wilcoxon rank sum test, Chi-square test, *t*-test, or Fisher’s exact test. The pain score, EQ-5D score, and coldness of the leg over time in each group and between groups were compared using the *t*-test, Mann-Whitney’s *U*-test, and Wilcoxon rank sum test. A *p*-value less than 0.05 was considered statistically significant.

## 3. Results

Of the 87 patients assessed for eligibility, three were excluded due to insufficient follow up data. Thus, a total of 84 patients were included in the analysis. The demographic and clinical characteristics of the patients are summarized in Table 1. Age, sex, body mass index, diagnosis, duration of pain, lumbar lesion level, and lesion severity did not differ statistically between the groups (Table 1). The surgery type, time after spinal surgery, number of spinal surgeries, and surgery range did not differ between the groups (Table 2). In the LSGB procedure, the LSGB level, side, and distance from surgery did not differ between the groups (Table 3). As shown in Table 3, the pain did not differ at T0 and was lower in the responder group at T1 and T4 (*P* < 0.001 and *P* = 0.000, respectively). The EQ-5D did not differ at T0, and the EQ-5D was lower in the responder group at T1 and T4 (*P* = 0.002, *P* = 0.004). Coldness of the leg did not differ between the groups. Table 4 shows the clinical outcomes of each group overtime. The pain difference between T1 and T0 and T4 and T0 was significantly different in both groups. In the responder group, the EQ-5D between T1 and T0 was significantly different (*P* = 0.046). In the non-responder group, EQ-5D and coldness of the leg scores did not differ over time (Table 4). Table 5 shows the clinical outcomes over time between the groups. The pain difference between T1 and T0 and T4 and T0 was significantly higher in the responder group (*P* < 0.001, *P* < 0.001). Moreover, the EQ-5D difference between T1 and T0 was significantly higher in the responder group than in the non-responder group (*P* = 0016). Coldness of the leg over time did not differ between the groups (Table 5). No serious adverse events occurred in either of the groups.

**TABLE 1:** Demographic and clinical characteristics of the patients.

	Patients (*n* = 84)	Responder (*n* = 41)	Non-responder (*n* = 43)	*P*-value
Age (years)	67.2 ± 10.2	65.8 ± 11.3	68.5 ± 9.0	0.296
Sex				0.801
Male	36 (42.9%)	17 (41.5%)	19 (44.2%)	
Female	48 (57.1%)	24 (58.5%)	24 (55.8%)	
Body mass index (kg/m^2^)	24.3 ± 2.7	24.5 ± 2.6	24.2 ± 2.9	0.729
Diagnosis				0.886
Spinal stenosis	65 (77.4%)	32 (78.1%)	33 (75.7%)	
HNP	19 (22.6%)	9 (22.0%)	10 (23.3%)	
Duration of pain (y)				>0.999
<1	2 (2.4%)	1 (2.4%)	1 (2.3%)	
1–2	7 (8.3%)	3 (7.3%)	4 (9.3%)	
>2	75 (89.3%)	37 (90.2%)	38 (88.4%)	
Lumbar lesion level				0.131
2-3	11 (13.1%)	5 (12.2%)	6 (14.0%)	
L3-4	26 (31.0%)	13 (31.7%)	13 (30.2%)	
L4-5	31 (36.9%)	19 (46.3%)	12 (27.9%)	
L5-S1	16 (19.1%)	4 (9.8%)	12 (27.9%)	
Lesion severity				0.751
Mild	21 (25.0%)	9 (22.0%)	12 (27.9%)	
Moderate	39 (46.4%)	19 (46.3%)	20 (46.5%)	
Severe	24 (28.6%)	13 (31.7%)	11 (25.6%)	

Values are mean ± SDs or numbers (percentages). HNP, herniated nucleus pulposus. The *P*-value for Chi-square test or Fisher’s exact test was set at 0.05.

**TABLE 2:** Surgical characteristics of patients.

	Patients (*n* = 84)	Responder (*n* = 41)	Non-responder (*n* = 43)	*P*-value
Surgery type				0.979
PLIF	44 (52.4%)	21 (51.2%)	23 (53.5%)	
Laminectomy	32 (38.1%)	16 (39.0%)	16 (37.2%)	
Discectomy	8 (9.5%)	4 (9.8%)	4 (9.3%)	
Time after spinal surgery				0.229
≤2 year	25 (29.8%)	14 (34.1%)	11 (25.6%)	
>2 year	59 (70.2%)	27 (65.9%)	32 (74.4%)	
Number of spinal surgeries	1.5 ± 0.7	1.4 ± 0.7	1.5 ± 0.7	0.302
Surgery range (level)				0.063
1	53 (63.1%)	26 (63.4%)	27 (62.8%)	
2	23 (27.4%)	8 (19.5%)	15 (34.9%)	
3	4 (4.8%)	4 (9.8%)	0 (0.0%)	
4	4 (4.8%)	3 (7.3%)	1 (2.3%)	

Values are mean ± SDs or numbers (percentages). PLIF, posterior lumbar interbody fusion. The *P*-value for Chi-square test or Fisher’s exact test was set at 0.05.

**TABLE 3:** Fluoroscopic data of lumbar sympathetic ganglion block and clinical outcomes over time.

	Patients (*n* = 84)	Responder (*n* = 41)	Non-responder (*n* = 43)	*P*-value
Block level				0.421
1 level	51 (60.7%)	22 (53.7%)	29 (67.4%)	
2 level	22 (26.2%)	13 (31.7%)	9 (20.9%)	
3 level	11 (13.1%)	6 (16.6%)	5 (11.6%)	
Block side				0.082
Left/Right/Both	21/20/43	12/13/16	9/7/27	
Block level distance from surgery				0.063
0 level	50 (59.5%)	23 (56.1%)	27 (62.8%)	
1 level	28 (33.3%)	2 (4.9%)	4 (9.3%)	
≥2 level	6 (7.1%)	16 (39.0%)	12 (27.9%)	
**Pain (NRS)**				
T0	7.0 ± 2.0	7.2 ± 1.7	6.7 ± 2.2	0.281
T1	6.1 ± 2.3	4.9 ± 2.0	7.3 ± 2.1	<0.001
T4	6.3 ± 2.1	5.4 ± 1.8	7.2 ± 2.1	0.000
**EQ-5D**				
T0	14.6 ± 4.0	14.4 ± 4.2	14.7 ± 3.9	0.728
T1	14.3 ± 3.4	13.1 ± 2.9	15.4 ± 3.6	0.002
T4	14.1 ± 3.2	13.1 ± 2.9	15.1 ± 3.3	0.004
**Coldness of legs (NRS)**				
T0	4.5 ± 3.5	4.6 ± 3.3	4.4 ± 3.7	0.877
T1	4.2 ± 3.1	4.1 ± 2.6	4.4 ± 3.6	0.462
T4	4.4 ± 3.1	4.5 ± 2.9	4.4 ± 3.3	0.818

Values are mean ± SDs or numbers (percentages). NRS, numerical rate scale; EQ-5D, EuroQol measure of health outcome; T0, before block; T1, 1 week after block; T4, 4 weeks after block, The *P*-value for Mann-Whitney’s *U*-test or *t*-test was set at 0.05.

**TABLE 4:** Clinical outcomes over time within each groups.

	Responder (*n* = 41)	*P*-value	Non-responder (*n* = 43)	*P*-value
**Pain (NRS)**				
T1 vs. T0	5.0 (4.0–6.0) vs. 7.0 (6.0–7.0)	<0.001	8.0 (7.0–9.0) vs. 7.0 (5.0–8.0)	<0.001
T4 vs. T1	5.0 (4.0–7.0) vs. 5.0 (4.0–6.0)	0.267	8.0 (6.0–8.0) vs. 8.0 (7.0–9.0)	>0.999
T4 vs. T0	5.0 (4.0–6.0) vs. 7.0 (6.0–7.0)	<0.001	8.0 (6.0–8.0) vs. 7.0 (5.0–8.0)	0.038
**EQ-5D**				
T1 vs. T0	13.0 (11.0–14.0) vs. 14.0 (12.0–18.0)	0.046	15.0 (13.0–18.0) vs. 15.0 (12.0–18.0)	0.348
T4 vs. T1	13.0 (12.0–15.0) vs. 13.0 (11.0–14.0)	>0.999	15.0 (12.0–18.0) vs. 15.0 (13.0–18.0)	>0.999
T4 vs. T0	13.0 (12.0–15.0) vs. 14.0 (12.0–18.0)	0.156	15.0 (12.0–18.0) vs. 15.0 (12.0–18.0)	0.933
**Coldness of leg (NRS)**				
T1 vs. T0	4.0 (2.0–6.0) vs. 5.0 (2.0–7.0)	0.177	5.0 (0.0–8.0) vs. 5.0 (0.0–8.0)	>0.999
T4 vs. T1	5.0 (3.0–7.0) vs. 4.0 (2.0–6.0)	0.727	5.0 (0.0–7.0) vs. 5.0 (0.0–8.0)	>0.999
T4 vs. T0	5.0 (3.0–7.0) vs. 5.0 (2.0–7.0)	>0.999	5.0 (0.0–7.0) vs. 5.0 (0.0)	>0.999

Values are median (IQR), IQR, interquartile range; NRS, numerical rate scale; EQ-5D, EuroQol measure of health outcome; T0, before block; T1, 1 week after block; T4, 4 weeks after block. The *P*-value for Mann-Whitney’s *U*-test or Wilcoxon signed rank test was set at 0.05.

**TABLE 5:** Clinical outcomes over time between two groups.

	Patients (*n* = 84)	Responder (*n* = 41)	Non-responder (*n* = 43)	*P*-value
**Pain (NRS)**				
T1–T0	0.0 (–2.0 to 0.0)	–2.0 (–3.0 to –1.0)	0.0 (0.0 to 1.0)	< 0.001
T4–T1	0.0 (–1.0 to 1.0)	0.0 (–1.0 to 2.0)	0.0 (0.0 to 0.0)	0.431
T4–T0	0.0 (–2.0 to 1.0)	–2.0 (–3.0 to 0.0)	0.0 (0.0 to 1.0)	< 0.001
**EQ-5D**				
T1–T0	0.0 (–3.0 to 2.0)	–1.0 (–4.0 to 0.0)	1.0 (–2.0 to 3.0)	0.016
T4–T1	0.0 (–2.0 to 1.0)	0.0 (–2.0 to 2.0)	0.0 (–2.0 to 1.0)	> 0.999
T4–T0	–1.0 (–3.0 to 2.0)	–1.0 (–4.0 to 0.0)	1.0 (–2.0 to 3.0)	0.122
**Coldness of leg (NRS)**				
T1–T0	0.0 (–1.0 to 0.0)	0.0 (–2.0 to 0.0)	0.0 (0.0 to 0.0)	0.114
T4–T1	0.0 (–1.0 to 1.0)	0.0 (–1.0 to 1.0)	0.0 (0.0 to 1.0)	0.948
T4–T0	0.0 (–2.0 to 1.0)	0.0 (–2.0 to 2.0)	0.0 (–1.0 to 1.0)	0.948

Values are median (IQR), IQR, interquartile range; NRS, numerical rate scale; EQ-5D, EuroQol measure of health outcome; T0, before block; T1, 1 week after block; T4, 4 weeks after block. The *P*-value for Mann-Whitney’s *U*-test or Wilcoxon signed rank test was set at 0.05.

## 4. Discussion

In the present study, we compared pain and quality of life in patients with FBSS who responded and did not respond to LSGB. LSGB reduced pain at all-time points. Patients who showed ≥ 50% reduction in pain at 1 week had improved quality of life simultaneously. However, patients who showed < 50% reduction in pain at 1 week had no improvement in quality of life. LSGB did not influence the coldness of the leg.

Chronic FBSS patients have pain, disability, insomnia, anxiety, and/or analgesic dependency (18). Proper pain management is needed to improve physical function and quality of life. Even with surgical treatment, adequate pain relief is not achieved in up to 30% patients after one back surgery and up to 70% patients after repeat surgery (1). FBSS remains difficult to manage due to its lack of precise pathophysiology and complexity of causes, and various clinical symptoms (18). In FBSS management, level one treatment includes pharmacological and non-pharmacological therapy, such as acupuncture and physiotherapy (18, 19). Level two treatment is recommended when level one treatment is unsuccessful; it includes selective root block, other spinal injections, and epidural adhesiolysis (18, 20). Manchikanti et al. recommend caudal epidural injection and epidural adhesiolysis for long-term improvement in FBSS (20). In FBSS after discectomy, caudal and transforaminal epidural steroid injection reduced pain and disability, and transforaminal injection was more effective in reducing disability at the 3-week follow-up (21). However, evidence of the long-term efficacy of epidural block remains insufficient. The magnitude of epidural scar tissue after spine surgery is related to pain intensity and limits the efficacy of epidural block (4). Inflammation of nerve roots and scaring can lead to radicular pain, and 20–36% of FBSS cases are associated with progressive epidural fibrosis (4). Even after epidural adhesiolysis, the pain can persists due to ongoing multiple pathophysiologic factors (20). Various causes, including mechanical tethering of nerve roots by underlying discs and pedicle, blood flow disturbances, and expression of pro-inflammatory cytokines causing dorsal root ganglion irritation trigger painful responses (20). Chronic neuropathic pain in FBSS is associated with central sensitization and impaired autonomic tone by sympathetic prevalence, which modulate the response to pain (22, 23). Sympathetic block has been used to alleviate sympathetic prevalence in numerous pain conditions, including neuropathic pain, vascular pain, and visceral pain (10, 13, 14). It disrupts pain perception by interrupting the pain signal that sympathetic nerves send to the brain (13). Local anesthetic injection on the lumbar sympathetic ganglions provide pain relief in the lower extremities (13). The enrolled patients in our study experienced refractory pain, even with spinal injections, before LSGB. A single shot of LSGB relieved the lumbosacral pain for 4 weeks. Pain reduction ≥ 50% improved quality of life. Therefore, we can suspect that the pain source in FBSS is significantly related to lumbar sympathetic ganglions, and substantial pain reduction is needed to improve quality of life. Additionally, the improvement in quality of life was shown only at 1 week, but not at 4 weeks. We suspect that considerable pain reduction leads to improved functional outcomes; therefore, further studies are needed to determine the proper cut-off value for pain change and quality of life change.

This study has several limitations. First, we did not follow up with the patients after 4 weeks; therefore, we did not confirm the long-term effects of LSGB. Second, we checked the accuracy of LSGB by confirming the spread of contrast media using fluoroscopy. Concurrent measurement of temperature changes in the leg is required for accuracy control. Third, we did not record changes in analgesic consumption during the follow-up period. Fourth, during LSGB, there was a possibility of spinal nerve block by the spreading of injectate *via* sympathetic rami-communicantes, which may influence pain severity. Fifth, the relatively small sample size may have resulted in imprecise estimates. Lastly, since there was no control group, no causal inferences can be made from these findings.

## 5. Conclusion

For patients who were responsive, LSGB is an effective method for treating pain in FBSS for a 4-week duration. Significant pain reduction may improve quality of life at 1-week later. Further studies are needed to achieve longer-lasting effects using different doses or injectate or repeated injections of local anesthetics for LSGB in FBSS. In addition, combinations of LSGB and other spinal interventions are required to determine their effects on clinical outcomes.

## Data availability statement

The original contributions presented in this study are included in the article/supplementary material, further inquiries can be directed to the corresponding author.

## Ethics statement

This study was approved by our departmental Ethics Committee (Catholic University of Korea: KC20RISI0917, Samsung Medical Center: SMC 2022-04-036) and registered with Clinical Research Information Service of the Korea National Institute of Health (http://cris.nih.go.kr/cris/index.jsp, ref: KCT0007236). Written informed consent for participation was not required for this study in accordance with the national legislation and the institutional requirements.

## Author contributions

JL and HP: conceptualization, data curation, formal analysis, investigation, methodology, and writing – review and editing. WS, JK, SY, HR, CK, and SK: data curation and investigation. All authors contributed to the article and approved the submitted version.

## Footnotes

1
http://cris.nih.go.kr/cris/index.jsp
